# Understanding Knowledgeable Workers’ Behavior Toward COVID-19 Information Sharing Through WhatsApp in Pakistan

**DOI:** 10.3389/fpsyg.2020.572526

**Published:** 2020-10-07

**Authors:** Talat Islam, Khalid Mahmood, Misbah Sadiq, Bushra Usman, Sheikh Usman Yousaf

**Affiliations:** ^1^Institute of Business Administration, University of the Punjab, Lahore, Pakistan; ^2^Department of Information Management, Faculty of Economics and Management Sciences, University of the Punjab, Lahore, Pakistan; ^3^Department of Economics and Finance, College of Economics and Management, Al Qasimia University, Sharjah, United Arab Emirates; ^4^School of Management, Forman Christian College, Lahore, Pakistan; ^5^Hailey College of Commerce, University of the Punjab, Lahore, Pakistan

**Keywords:** theory of planned behavior, COVID-19, information sharing behavior, social media, developing country, theory of prosocial behavior, theory of use and gratification

## Abstract

Using social media through mobile has become a major source of disseminating information; however, the motivations that impact social media users’ intention and actual information-sharing behavior need further examination. To this backdrop, drawing on the uses and gratifications theory, theory of prosocial behavior, and theory of planned behavior, we aim to examine various motivations toward information-sharing behaviors in a specific context [coronavirus disease 2019 (COVID-19)]. We collected data from 388 knowledgeable workers through Google Forms and applied structural equation modeling to test the hypotheses. We noted that individuals behave seriously toward crisis-related information, as they share COVID-19 information on WhatsApp not only to be entertained and seek status or information but also to help others. Further, we noted norms of reciprocation, habitual diversion, and socialization as motivators that augment WhatsApp users’ positive attitude toward COVID-19 information-sharing behavior.

## Introduction

A decade ago, information about crises was first informed by the affected ones through mobile phones, then were reported on social media (Oh et al., 2011). Nowadays, social media has become a major and rapid source of improvising, communicating, and distributing information during crises (Zhao et al., 2016). This is because social media has shown a great potential to respond to affected people during crises. However, there remained a criticism on the accuracy and quality of the information provided through social media by the volunteers (Alexander, 2014). For instance, at the early stage of tragedy or crisis, complete information about crises may not be available, and if in such situations social media users keep on posting and reposting inaccurate information, these could result in serious damages. Indeed, social media is a quick source of distributing information or rumors compared with traditional media (Tripathy et al., 2013). In fact, while searching “false news about earthquake,” one can find millions of fake news about the incident posted by citizens, and most of the news is there to create more panic about another imminent earthquake (Tanaka et al., 2013).

It does not necessarily mean that social media is only a source to spread false information during crises; in fact, it can be used as a channel to combat rumors. Zhao et al. (2016) noted that social media users first authenticate and then broadcast crises-related information. Similarly, Bird et al. (2012) also noted social media users’ positive attitudes toward crises-related information sharing. In March 2011, when Japan was hit by an earthquake tsunami, social media (Twitter) was actively involved, as Stirratt (2011) noted 49% of the circulated information was either positive or somewhat positive, whereas only 7% of the information was negative or somewhat negative about the emergency response. The world is facing a similar kind of problem because of the new pandemic [coronavirus disease 2019 (COVID-19)]. The issue (COVID-19) is still new with lots of rumors on social media.

In December 2019, Hubei province in China captured the world’s attention when pneumonia (lung disease) caused by a coronavirus emerged in Wuhan. The city of Wuhan is located in central China and is a key industrial and transportation hub with over 11 million population. At the beginning, it was believed that severe acute respiratory syndrome coronavirus 2 (SARS-CoV-2) is typically not transmissible to humans, as it has its origin rooted back to animals. However, in case of SARS, this virus is transmissible from animals to humans and humans to other humans. The severity of virus can be estimated by the fact that it took 3 months to reach the first 100,000 cases and only 12 days for another 100,000 cases (WHO, 2020).

Numerous misinformation is reported on several social media platforms regarding cure, prevention, outcomes, and etiology of the disease (Sandhya, 2020). Although these rumors are masking health behaviors, however, promoting erroneous practices is not only spreading the virus but also causing poor mental and physical health. For example, in India, a father of three kids was diagnosed with COVID-19 who then committed suicide (Joe, 2020). Similarly, after hearing about chloroquine (a drug primarily used to treat malaria), as a powerful drug to treat COVID-19 on media, several Nigerians were reported overdosed by their health minister (Busari and Adebyo, 2020). Similarly, the news of lockdown created panic regarding stationeries and groceries, which unbalanced demand–supply gaps and disrupted the supply chain in many countries (Spencer, 2020). These rumors largely affected individuals’ psychological and physical health, thereby generating the need to study what motivates social media users to share such information.

Ji et al. (2014) used rumor dynamic theory and developed an anti-rumor model. Similarly, Tripathy et al. (2011) and Tripathy et al. (2013) also developed anti-rumor models (i.e., neighborhood, beacon, and delayed start models). These models were developed for social media through a technological perspective and thus are very complex to understand for a layman. However, studies suggesting anti-rumor models from a social–psychological perspective are scarce. For example, Zhao et al. (2016) developed a norm activation model based on the theory of planned behavior to understand social media users’ information-sharing behavior, while Chen et al. (2018) extended this model by examining motivational factors toward such behaviors and suggested future researchers to identify more factors. In addition, past studies have highlighted the role of social media (mostly Facebook or Twitter) toward dissemination of crisis-related information (Tanaka et al., 2013; Zhao et al., 2016; Chen et al., 2018). However, studies on the factors that motivate social media users (WhatsApp) to share such information are limited. To fill in this gap, we selected WhatsApp users because statistics show that 29 million WhatsApp messages are sent every minute in Pakistan (Khan, 2020).

Moreover, past studies have identified entertainment, “individual’s desire to experience emotions through online participation” (Park et al., 2009), information seeking, “seeking for information as a consequence of a need to satisfy some goal” (Lee and Ma, 2012), socialization, “talking with others to achieve a sense of community and peer support on the particular topic of the group” (Karnik et al., 2013), status seeking, “maintaining personal status, as well as of their friends, through the online group participation” (Malik et al., 2016), habitual diversion, “entertaining activity as an escape from reality or routine” (Krause et al., 2014), and norms of reciprocity, “repaying in kind what others have done for us” (Chen et al., 2018), as motivators for information-sharing behavior on social media. However, how these motivators work holistically during crises (COVID-19 in this study) and the benefits associated with these need to be shed light. Therefore, we aim at extending past studies by examining the roles of socialization, status seeking, norms of reciprocity, habitual diversion, information seeking, and entertainment (motivational factors) of WhatsApp users’ attitudes toward COVID-19 information-sharing behavior. We used Batson’s (1990) theory of prosocial behavior (TPSB), Ajzen’s (1991) theory of planned behavior (TPB), and Katz et al.’s (1974) uses and gratifications theory (U&G) to develop a novel model toward social media sharing behavior of knowledgeable workers. In simple words, our study aims to examine:

(1) The role of socialization, status seeking, habitual diversion, information seeking, norms of reciprocity, and entertainment toward COVID-19 information-sharing behavior through WhatsApp (supporting from TPSB and U&G).

(2) How these factors affect the actual behaviors (TPB)

### Uses and Gratifications Theory

According to U&G, individuals fulfill their gratifications by selecting specific media over alternatives. Literature has suggested U&G as the utmost significant theory that explains the determinants and meaning of social media users’ behavior in the field of communication studies (Malik et al., 2016). Researchers started using U&G in explaining and identifying the motivations behind the use of traditional media. However, with the passage of time, traditional media was replaced by internet, which changed individuals’ behavior of using social media. Few of the studies have used U&G to examine the users’ motivations of using social media, such as Twitch, Snapchat, Instagram, Twitter, WeChat, and Facebook (Phua et al., 2017; Sjöblom et al., 2017; Chen et al., 2018). Kim and Yang (2017) argued that social media users use “share,” “comments,” “care,” and “like/dislike” as communication behaviors. Among these, “like/dislike” and “care” are driven by affect, whereas “comment” is driven by cognition. However, “share” is driven by both cognition and affection.

Krause et al. (2014) suggested that individuals highly motivate, involve, and devote when contributing something *via* social media, and their sharing depends upon communal incentive and self-interest (Fu et al., 2017). Indeed, content such as music (Krause et al., 2014), links (Baek et al., 2011), pictures (Malik et al., 2016), information regarding health (AlQarni et al., 2016), news (Lee and Ma, 2012), and crises-related information (Chen et al., 2018) matters while sharing on social media. Malik et al. (2016) identified information seeking, status seeking, and habitual diversion as gratification among 368 Facebook users while posting photos. Chen et al. (2018) identified norm of reciprocity, habitual diversion, and status seeking motivators for sharing crises-related information on WeChat. AlQarni et al. (2016) analyzed 1,551 Facebook posts on diabetes mellitus from the Arabic world to understand users’ gratification. They concluded that most of the users post their personal experiences to create awareness as norms of reciprocity. Park et al. (2009) noted that most of the information-sharing activities on social media (Facebook) take place through group applications. They noted that most of the students use social media to seek information about civic activities, status seeking, and socializing, instead of political activities. Lee and Ma (2012) studied 203 students and identified that socialization and status seeking positively influence while entertainment and information seeking insignificantly associated with their intention to share information. According to Chen et al. (2018), factors that motivate social media users to share crises-related information need further attention. Therefore, we aim to examine how previously examined motivations (getting entertainment, seeking information, habitual diversion, status seeking, socialization, and norms of reciprocity) for information-sharing behaviors on social media can make a difference during COVID-19 outbreak with the assumption that getting entertainment may negatively affect said behaviors. According to Zhao et al. (2016), social media users may behave with maturity regarding sharing crises-related information. More specifically, Chen et al. (2018) studied 365 WeChat users and noted a negative influence of entertainment on attitudes toward behavior for crises-related information. We extend existing literature in two ways. First, past studies have examined these motivators with information-sharing intention (Park et al., 2009; Lee and Ma, 2012); we extend these studies and attempt to understand these motivators through TPB. Therefore, we examined these motivators’ influence on attitudes toward behavior, subjective norms (SN), and perceived behavioral control (PBC). The motivators, i.e., getting entertainment, seeking information, habitual diversion, status seeking, socialization, and norms of reciprocity, help individuals to evaluate their favorable or non-favorable behaviors (PBC) (Park et al., 2009; Malik et al., 2016; Chen et al., 2018). Whereas status seeking and socialization can also affect individuals PBC (*an individual’s perception about ease or difficulty to perform a behavior*) and SNs [*an individual’s perception about whether his/her near ones (e.g., teachers, friends, peers, spouse, and parents) want him/her to behave in a specific manner*], given that individuals around us impact our beliefs about favorable situations. Specifically, we aim to examine whether the gratification of sharing COVID-19 information on social media identified by literature (in isolation) can impact WhatsApp users’ attitudes toward information-sharing behavior, SNs, and PBC. Thus, we may hypothesize:

H1: Getting entertainment (a) has a negative impact, whereas seeking information (b), habitual diversion (c), status seeking (d), and socialization (e) have a positive impact, on WhatsApp users’ attitudes toward COVID-19 information-sharing behavior.

H2: Seeking status (a) and socialization (b) have a positive impact on WhatsApp users’ subjective norms about COVID-19 information-sharing behavior.

H3: Seeking status (a) and socialization (b) have a positive impact on WhatsApp users’ perceived behavioral control toward COVID-19 information-sharing behavior.

### Theory of Prosocial Behavior

We extend the literature by arguing that, in addition to gratification, individuals may voluntarily share COVID-19 information on WhatsApp for prosocial purposes (i.e., TPSB). According to Sanstock and Topical (2007), prosocial behavior includes obeying rules, cooperating, donating, sharing, helping, and complying to socially acceptable behaviors. However, social psychologists posit a different perspective behind individuals’ prosocial behavior on social media. Morozov (2010) argued that social media users lack strong bonding, therefore, it may not be an essential platform for prosocial behavior, called “Slacktivism.” In contrast, while studying Facebook and Twitter, Fatkin and Lansdown (2015) noted a significant association between exposure to social media and prosocial behavior. For example, Michelle Sollicito created a page “Snowed Out Atlanta” on Facebook to help people after sensing traffic gridlock, as a result, many open pages and groups were created to help people in snow disasters. Likewise, a page “blood donations of Hailey College of Banking and Finance” created many other open groups to help those who need blood in the country. Similarly, the concept of Black Friday by Americans was adopted by many other countries in the world (Fatkin and Lansdown, 2015). These findings show that, despite weak ties among users, social media can be a source of prosocial helping behaviors.

While exploring prosocial behavior, Eisenberg et al. (1998) identified social status, egoistic concerns, perceived fairness system, empathy toward others’ welfare, and reciprocity as the motivations behind such behaviors. Pai and Tsai (2016) argued that norms of reciprocity may be the key motivating factor that impact individuals’ information-sharing behavior. Norms of reciprocity is a universal norm that individuals must pay back to the one who helped them at the time of need (Gouldner, 1960). Literature is mixed while applying the concept of reciprocity on information-sharing behavior on social media. For example, Wasko and Faraj (2000) noted a negative, Wiertz and De Ruyter (2007) noted an insignificant, while Chang and Chuang (2011) noted a positive and significant association of norms of reciprocity with individuals’ information-sharing behavior on social media. It can be inferred that the association between norms of reciprocity and information-sharing behavior may depend on the context and conditions (Pai and Tsai, 2016). Following the same, we argue that in case of crises, WhatsApp users consider it their responsibility to share accurate and updated information to benefit sufferers, thus we may hypothesize:

H4: Norms of reciprocity have a positive impact on WhatsApp users’ attitudes (a), subjective norms (b), and perceived behavioral control (c) toward COVID-19 information-sharing behavior.

### Theory of Planned Behavior

According to Ajzen (2011), individuals’ behavior is dependent upon their belief about controlling their behavior, perception about their near ones that they want them to perform a certain behavior, and/or they have a favorable attitude toward that behavior. Thus, TPB elucidates the inspiring and informational influence on individuals’ behaviors. Researchers have been using TPB in the field of computers since the 1980s. However, few of the researchers have used this theory in explaining users’ online behaviors, such as service and shipping usage (Lu et al., 2007), watching video (Cha, 2013), and shopping (Cheng and Huang, 2013). Later, researchers start using TPB in exploring individuals’ behavior using social media such as privacy protection (Taneja et al., 2014), combating rumor (Zhao et al., 2016), crises information sharing (Chen et al., 2018), and location disclosure (Chang and Chen, 2014). Zhao et al. (2016) inculcates that social media-related behaviors can best be explained with the help of TPB. Given that, we aim to extend these studies by examining WhatsApp user’s behavior during the COVID-19 pandemic through TPB. As discussed earlier, TPB explains an individual’s behavioral intention (BI) through three aspects, i.e., “*attitude toward behavior, subjective norms, and perceived behavioral control*.” According to Ajzen (2011), individuals first evaluate their behavior (favorable or not favorable) to develop their BI, called attitude toward behavior (ATB). We argue that COVID-19 information-sharing attitude impacts social media users’ sharing intention. Following the same, we hypothesize:

H5: A positive attitude toward COVID-19 information sharing has a positive impact on WhatsApp users’ intention to share COVID-19 information.

As per TPB, the second aspect that influences BI is SNs. SN refers to an individual’s perception about whether his/her near ones (e.g., teachers, friends, peers, spouse, parents, etc.) want him/her to behave in a specific manner (Ajzen, 2011). In simple words, SN is an individual’s perception about consent or condemnation of his behaviors by the majority (Amjad and Wood, 2009). Chang et al. (2014) noted that an individual’s 63% of the variance of gameplay intentions was explained by SNs. Similarly, Bai et al. (2014) noted an individual’s 57% of the intentions to continue hygienic food-handling behavior is explained by SNs. Particular to social media, Chen et al. (2018) also found SNs positively impacting on individuals’ intention of sharing crises-related information. Therefore, we may hypothesize:

H6: Subjective norms have an impact on WhatsApp users’ intention to share COVID-19 information.

According to TPB, PBC is the third aspect that impacts BI. This aspect varies across situations because it is an individual’s perception about ease or difficulty to perform a behavior. Therefore, individuals when perceiving favorable situations would behave accordingly. Ajzen (2011) inculcates that PBC also has a tendency to impact individual’s actual behaviors (AB). In particular, individuals have multiple sources to share information; however, we aim to examine how PBC predict WhatsApp users’ actual and BI to share COVID-19 information. Thus, we hypothesize:

H7: Perceived behavioral control has a positive impact on WhatsApp users’ behavioral intention (a) and actual behavior (b) of sharing COVID-19 information.

Literature has suggested that individual’s BI positively affects their ABs in microblogging (Jiang et al., 2016) and transportation (Bamberg et al., 2007). Whereas others have identified a mixed result studying solar energy usage (Hai et al., 2017) and combating rumor (Zhao et al., 2016) and electronic waste (Echegaray and Hansstein, 2017). Thus, there is a need to further examine the association between BI and AB. We aim to examine whether intention to share COVID-19 information affects individuals’ AB toward information sharing or not by hypothesizing:

H8: Behavioral intention has a positive impact on WhatsApp users’ behavior of sharing COVID-19 information.

## Materials and Methods

### Sample and Procedure

We collected data from the students of MBA executive because of the following reasons. First, we wanted to understand the behaviors of well-educated people toward COVID-19. Higher Education Commission of Pakistan has authorized universities that an applicant must have 16 years of education with a minimum of 2 years of work experience to be enrolled in MBA executive (which served the purpose). Second, although English is considered as the official language, still many of the employees remained unable to understand English (Raja et al., 2004; Islam et al., 2019, 2020a,b), thus educated people were selected as they can better respond to the questionnaires in English. Finally, during the lockdown, data collection was difficult in real settings.

We conducted an online survey where a link was shared on the WhatsApp groups of executive students. The students were noted to disseminate COVID-19-related information on these groups on a frequent basis. Further, few of the students or their family members were COVID-19 positive. The respondents were well explained about the purpose of this study and were ensured about the anonymity of their responses. Within 15 days, we received 394 responses out from 420 students. The data on all variables were collected from the same respondent; therefore, we followed the instructions of Podsakoff et al. (2012) to cope with the issue of common method variance (CMV). In addition, we also examined Harman’s single-factor test, and a single factor was found to have no more than 50% variance. The test supported the conclusion that CMV is absent.

We consider age, gender, qualification, and sector as control variables as these can have effects on respondents’ attitudes and behaviors (Davis et al., 2019; Ahmad et al., 2020). According to gender, 84.5% (*n* = 328) of the respondents were male and 15.5% (*n* = 60) of the respondents were female, which represent the male-dominant culture of the country (Islam et al., 2020b). According to age, 46.4% (*n* = 180) of the respondents were between 31 and 40 years, 35.6% (*n* = 138) were less than 30 years, 12.4% (*n* = 48) were between 41 and 50 years, and only 5.7% (*n* = 22) were above the age of 50 years. Based on sector, 64.7% (*n* = 251) of the respondents were from the manufacturing sector, while 35.3% (*n* = 137) were from the service sector. Interestingly, 33.2% (*n* = 129) of the respondents were habitual WhatsApp users for at least 2 h/day, 30.4% (*n* = 118) use WhatsApp for 3 h/day, 19.3% (*n* = 75) use WhatsApp for 1 h/day, and 17.0% (*n* = 66) were using WhatsApp for more than 3 h/day.

### Measures

We adapted questionnaires from the past studies and modified them according to the situation (COVID-19). Respondents responded using a five-point Likert scale (see Appendix A). We used six factors (i.e., norms of reciprocity, socialization, status seeking, habitual diversion, information seeking, and entertainment) about the reasons of participating in online discussions. Among these, questionnaires on four factors (i.e., self-status seeking, socialization, information seeking, and entertainment) comprised of three items for each factor were adapted from the study of Park et al. (2009), who reported their reliability ranges between 0.81 and 0.87. These factors were also validated by Chen et al. (2018) in the Southeast Asian context. Using the same factors, we noted its values of Cronbach’s alpha ranges between 0.70 and 0.82. We adapted another three-item scale of habitual diversion from the study of Malik et al. (2016) and noted 0.71 as the value of its reliability. Finally, norms of reciprocity were measured through a three-item scale of Pai and Tsai (2016), and we noted 0.73 as the value of its reliability.

Information about (SN, ATB, PBC, AB, and BI was obtained through Ajzen’s (1991) three-item scale for each. These scales have been validated by Oh et al. (2013), Han (2015), Zhao et al. (2016), and Chen et al. (2018). We noted 0.70, 0.79, 0.83, 0.86, and 0.83 as the values of its reliability, respectively (see Appendix A).

### Statistical Analyses

We applied structural equation modeling (SEM) to test the hypotheses. The data were examined for the basis assumptions of SEM (e.g., missing values, outliers, normality, and multicollinearity). First, we conducted a confirmatory factor analysis (CFA) as we used validated scales. CFA was performed using AMOS version 24, applying maximum likelihood estimation. According to Mîndrilã (2010), in case of an ordinary scale, weighted least squares (WLS) parameter is best but only when data are asymmetric or show a high level of heteroskedasticity. The data for the study were examined for heteroskedasticity and found to be normally distributed; therefore, maximum likelihood method was used (Li, 2016). We followed Williams et al. (2009) for mode fit indices, Hair et al. (2018) for the values of factor loading, composite reliability, and average variance extracted, and Cronbach’s alpha. We then examined Pearson correlation to examine the strength of bivariate relationships among variables. Finally, we examined the structural model to test the hypotheses.

## Results

We examined the hypotheses through SEM using AMOS. Therefore, first, the data were examined to fulfill their basic assumptions (i.e., missing values, outliers, normality, and collinearity).

### Preliminary Analyses

The data (394 responses) were found to be free from missing values because they were collected through Google Forms and a condition of compulsory answer was applied. We applied Mahalanobis distance test at *P* < 0.01 to identify outliers; therefore, six were excluded (Hair et al., 2018). Regarding normality, the values of skewness and kurtosis (i.e., ±1 and ±3, respectively) were noted to be within range (Byrne, 2016). Finally, none of the correlation was found to be more than 0.85 (Table 1), which identifies the absence of collinearity in the data (Tabachnick and Fidell, 2019).

**TABLE 1:** Results of correlation, mean, and standard deviation.

**Variables**	**1**	**2**	**3**	**4**	**5**	**6**	**7**	**8**	**9**	**10**	**11**
(1) Norms of Reciprocity (NOR)	1										
(2) Socialization	0.59**	1									
(3) Status Seeking (SS)	0.55**	0.67**	1								
(4) Habitual Diversion (HD)	0.63**	0.60**	0.58**	1							
(5) Information Seeking (IS)	0.54**	0.58**	0.53**	0.51**	1						
(6) Entertainment	−0.44**	−0.30**	−0.38**	−0.40**	−0.42**	1					
(7) Subjective Norms (SN)	0.45**	0.33**	0.35**	0.43**	0.38**	−0.47**	1				
(8) Attitude Toward Behavior (ATB)	0.58**	0.43**	0.37**	0.48**	0.40**	−0.38**	0.31**	1			
(9) Perceived Behavioral Control (PBC)	0.55**	0.53**	0.50**	0.50**	0.49**	−0.55**	0.41**	0.53**	1		
(10) Actual Behavior (AB)	0.46**	0.33**	0.38**	0.40**	0.32**	−0.53**	0.49**	0.43**	0.53**	1	
(11) Behavioral Intention (BI)	0.56**	0.45**	0.47**	0.49**	0.44**	−0.59**	0.49**	0.47**	0.61**	0.63**	1
Mean	3.82	3.56	3.60	3.59	3.63	1.98	3.83	3.56	3.47	3.72	3.82
Standard Deviation	0.71	0.70	0.74	0.75	0.71	0.68	0.65	0.79	0.82	0.84	0.81

### Descriptive Statistics

The results of descriptive statistics are presented in Table 1. The mean values show that the respondents agreed about five factors [i.e., norms of reciprocity (3.82), socialization (3.56), status seeking (3.60), habitual diversion (3.59), and information seeking (3.63)], whereas respondents disagreed about entertainment (1.68) as the reason for participating in online discussions during the COVID-19 pandemic. Further, they also agreed on SNs (3.83), ATB (3.56), perceived behavioral control (3.47), actual behavior (3.72), and behavioral intention (3.82). Moreover, the values of Cronbach’s alpha of all the variables were also noted well above the standard value of 0.70 (Hair et al., 2018) (Appendix A). Further, we noted positive and significant correlations among variables used (*r* ranging between 0.32 and 0.67, *P* < 0.05), except entertainment as it was noted to have a negative correlation with other variables (*r* ranging between −0.30 and −0.59, *P* < 0.01).

### Structural Equation Modeling

We followed Anderson and Gerbing (1998), and SEM was applied in two stages where, first, CFA was conducted to examine the measurement model (11-factor model as all the factors were included while examining the measurement model) because scales used by us were adapted; second, the structural model was examined. We used “chi-square/degree of freedom (*x*^2^/*df ≤* 3.0), Tucker–Lewis index (TLI ≥ 0.90), comparative fit index (CFI ≥ 0.90), goodness-of-fit index (GFI ≥ 0.90), root mean residual (RMR ≤ 0.10), and root mean square error of approximation (RMSEA ≤ 0.08)” for model fit, as suggested by Williams et al. (2009) and found our model fit, i.e., *x*^2^/*df* (981.248/440) = 2.23, TLI = 0.90, CFI = 0.91, GFI = 0.90, RMR = 0.039, RMSEA = 0.056, and P-Close = 0.014. Further, we followed Hair et al. (2018) to examine loading (i.e., ≥0.50), average variance extracted (i.e., ≥0.50), and composite reliability (i.e., ≥0.60) and noted that our scales fulfilled the criteria (see Appendix A).

### Hypotheses Testing

**FIGURE 1:**
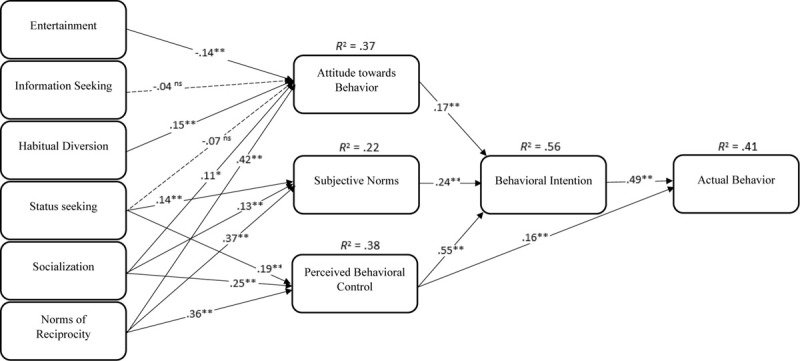
Structural model.

Results generated through the structural model (maximum likelihood parameter estimation) are presented in Figure 1 and Table 2. The structural model was found to be fit, i.e., *x*^2^/*df* (1,077.551/465) = 2.31, TLI = 0.92, CFI = 0.93, GFI = 0.92, RMR = 0.044, RMSEA = 0.058, and P-Close = 0.001. The values revealed that entertainment negatively impacts (β = −0.14, CR = −3.190, *P* = 0.001), habitual diversion (β = 0.15, CR = 3.447, *P* = 0.000), socialization (β = 0.11, CR = 2.497, *P* = 0.013), and norms of reciprocity (β = 0.42, CR = 9.563, *P* = 0.000) positively impact, whereas seeking information (β = −0.04, CR = 0.901, *P* = 0.368) and status seeking (β = −0.07, CR = −1.548, *P* = 0.122) insignificantly impact on WhatsApp users’ attitudes toward COVID-19 information-sharing behavior. These findings support H1a, H1c, H1e, and H4a and rejects H1b and H1d, respectively. The values further show that seeking status (β = 0.14, CR = 3.011, *P* = 0.003), socialization (β = 0.13, CR = 2.786, *P* = 0.000), and norms of reciprocity (β = 0.37, CR = 7.899, *P* = 0.000) were also noted to have a positive impact on WhatsApp users’ SNs about COVID-19 information-sharing behavior. These results support H2a, H2b, and H4b, respectively. Similarly, seeking status (β = 0.19, CR = 4.144, *P* = 0.000), socialization (β = 0.25, CR = 5.609, *P* = 0.000), and norms of reciprocity (β = 0.36, CR = 8.131, *P* = 0.000) were also noted to have a positive impact on WhatsApp users’ perceived behavioral control toward COVID-19 information-sharing behavior. These results support H3a, H3b, and H4c, respectively.

**TABLE 2:** Results of hypotheses testing.

**Hypotheses**	**Standardized β**	**CR**	**SE**	***P***	**Result**
Entertainment→Attitude Toward Behavior	–0.14	–3.190	0.046	0.001	H1a is accepted
Information Seeking→Attitude Toward Behavior	–0.04	0.901	0.045	0.368	H1b is rejected
Habitual Diversion→Attitude Toward Behavior	0.15	3.447	0.043	0.000	H1c is accepted
Status Seeking→Attitude Toward Behavior	–0.07	–1.548	0.043	0.122	H1d is rejected
Socialization→Attitude Toward Behavior	0.11	2.497	0.045	0.013	H1e is accepted
Norms of Reciprocity→Attitude Toward Behavior	0.42	9.563	0.043	0.000	H4a is accepted
Status Seeking→Subjective Norms	0.14	3.011	0.040	0.003	H2a is accepted
Socialization→Subjective Norms	0.13	2.786	0.042	0.000	H2b is accepted
Norms of Reciprocity→Subjective Norms	0.37	7.899	0.042	0.000	H4b is accepted
Status Seeking→Perceived Behavioral Control	0.19	4.144	0.044	0.000	H3a is accepted
Socialization→Perceived Behavioral Control	0.25	5.609	0.047	0.000	H3b is accepted
Norms of Reciprocity→Perceived Behavioral Control	0.36	8.131	0.046	0.000	H4c is accepted
→Attitude toward BehaviorBehavioral Intention	0.17	2.646	0.039	0.008	H5 is accepted
Subjective Norms→Behavioral Intention	0.24	6.425	0.042	0.000	H6 is accepted
Perceived Behavioral Control→Behavioral Intention	0.55	14.506	0.038	0.000	H7a is accepted
Behavioral intention→Actual Behavior	0.49	9.180	0.058	0.000	H8 is accepted
Perceived Behavioral Control→Actual Behavior	0.16	3.103	0.057	0.002	H7b is accepted

*CR represents *t*-value. SE, standard error; P, significance.*

The results further revealed that ATB (β = 0.17, CR = 2.646, *P* = 0.008), SNs (β = 0.24, CR = 6.425, *P* = 0.000), and perceived behavioral control (β = 0.55, CR = 14.506, *P* = 0.000) positively impact WhatsApp users’ intention to share COVID-19 information. Finally, perceived behavioral control (β = 0.16, CR = 3.103, *P* = 0.002) and behavioral intention (β = 0.49, CR = 9.180, *P* = 0.000) were also found to predict WhatsApp users’ actual behavior toward COVID-19 information. These results support H5, H6, H7a, H7b, and H8, respectively.

## Discussion

The aim of this study was to develop and understand a model about the motivations toward WhatsApp users’ COVID-19 information-sharing behavior in a developing country. We consider the framework of TPB and extend with the help of TPSB and U&G. We examined hypotheses on 388 responses collected during the COVID-19 pandemic through an online survey. Unlike past studies, the findings of this study are interesting. For example, past studies confirmed that most of the social media users (especially mobile) consider social media a source of entertainment (Leggatt, 2011). Tsang et al. (2014) noted that entertainment positively associated with users’ ATB. On the other hand, Lee and Ma (2012) identified an insignificant association between entertainment and information-sharing behavior. Further, Chen et al.’s (2018) findings revealed a negative association between entertainment and attitude toward information sharing. Similarly, information seeking and status seeking also show a mixed result. For example, Malik et al. (2016) identified that social media users share information (photos) for information seeking and status seeking, while Chen et al. (2018) identified a non-significant association of status seeking and information seeking with ATB. It can be inferred that motivating factors impact individuals’ information-sharing behavior differently in different contexts, i.e., situation, culture, etc. (Fu et al., 2017). Considering the situational factor (i.e., COVID-19), we noted that individuals do not share COVID-19 information on WhatsApp to be entertained. Precisely, individuals respond to crises with a serious attitude and try to disseminate authentic information (Chen et al., 2018). Contradicting previous studies, we further noted that information and status seeking does not motivate individuals toward information sharing during the COVID-19 pandemic. This may be because individuals primarily focus on the pandemic (crisis) and want to be assured before sharing the same information on social media as they prefer to combat rumors (Zhao et al., 2016). In line with literature, we also noted socialization, habitual diversion, and norms of reciprocity as motivating factors for “attitudes toward information-sharing behavior.”

Past studies have identified socialization, status seeking, and norms of reciprocity as the motivational factors for SNs; we identified the same for perceived behavioral control as well. This finding suggests that WhatsApp users use prosocial behaviors regarding sharing information, rather than being just rumor mills (Hjorth and Kim, 2011). This finding can be justified by arguing, although status seeking, and socializing is considered bad during the pandemic; still, the desire to connect with others to get helpful information overcomes the fear of information sharing. According to Kim (2014), individuals are prone to anxiety when they feel isolated or find themselves with lack of sufficient information. However, having themselves equipped with timely information may help them in getting out of the state of anxiety. Regarding norms of reciprocity, individuals consider it their responsibility to pay back to the society by sharing pandemic-related information on WhatsApp.

Finally, consistent with the TPB, we noted that ATB, SNs, and perceived behavioral control positively predict WhatsApp users’ behavioral intention and actual behavior toward COVID-19-related information. This finding suggests that WhatsApp users who feel an obligation have a positive attitude toward others and are more confident about sharing information and are more likely to be involved in sharing COVID-19 information with others. Replying to the contradictory results (Zhao et al., 2016), we identified behavioral intention as the predictor of actual behavior. Thus, TPB, U&G, and TPSB fit to understand WhatsApp users’ information-sharing behavior during the COVID-19 pandemic.

### Implications and Limitations

The findings of our study contribute theoretically and practically. First, most of the previous studies regarding information-sharing behavior have been conducted in western countries where Twitter or Facebook remained their prime focus. However, the prime focus of our study was to understand the individuals’ information-sharing behavior during COVID-19 in a non-western context. Second, past studies mostly have studied generic information-sharing behaviors (e.g., Zhao et al., 2016; Chen et al., 2018), whereas we examined the same in a specific context (COVID-19) and found contradictory results, which generated the need to further understand social media users’ information-sharing behavior along with their motivations. Third, as we investigated the relationship between motivations and information sharing, therefore, the findings of our study may likely benefit academicians, policy makers, and all other related stakeholders. Finally, our study extends the existing literature about information sharing in the field of behavioral research by combining TPSB, U&G, and TPB.

This study suggests practitioners to handle crises by understanding that educated individuals in developing countries are very serious regarding disseminating crises-related information. They do not share information to be entertained or seek status, but to be socialized as to alleviate their anxiety and tension by sharing crisis-related information (COVID-19 here). Further, educated social media users feel that it is their responsibility to share crisis-related information with others for their betterment and to combat rumors. Given that, healthcare professionals should release relevant and sufficient information on social media through different channels, such as WhatsApp, Twitter, Facebook, or Snapchat, etc. While doing so, disseminating misleading information may be prevented.

Despite implications, the study has few limitations. First, we collected data from highly educated individuals using WhatsApp, which may raise a question on its generalizability to other populations as the results might be different considering less educated individuals and other social media channels. Second, most of the respondents of this study were male, which may raise a question of gender bias results. Therefore, future researchers are suggested to have equal representation of both male and female participants. Third, the data on independent and dependent variables were collected from the same source, which may generate biased results; therefore, future researchers are suggested to collect data as dyads (i.e., user and his/her colleague). In addition, such data restrict the researchers to identify the exact direction. Fourth, there exists a gap in the measures used as some of the questions are about sharing COVID information, while others are about sharing authentic COVID information. Finally, we used motivations based on U&G and TPSB; future researchers are suggested to identify other unexplored motivations toward information-sharing behavior.

## Conclusion

Drawing upon the U&G, TPSB, and TPB, we examined a model to understand the motivations that impact social media (WhatsApp) users while sharing COVID-19 information. We noted that social media users do not share crises-related information to be entertained or for information seeking and status seeking. They behave with maturity and consider their responsibility to share authentic information during crises. The findings of this study suggest that healthcare professionals share relevant information on social media for further dissemination. Such policies would not only help victims in adopting accurate precautionary measures but also help to combat rumors.

## Data Availability Statement

The raw data supporting the conclusions of this article will be made available by the authors, without undue reservation.

## Ethics Statement

The studies involving human participants were reviewed and approved by Institute of Business Administration, University of the Punjab. Written informed consent for participation was not required for this study in accordance with the national legislation and the institutional requirements.

## Author Contributions

TI developed the manuscript, collected the data, and conducted the analysis. KM initiated the idea. MS helped in incorporating suggested changes, while BU and SY gave the manuscript a final proofread. All the authors contributed to the article and approved the submitted version.

## Conflict of Interest

The authors declare that the research was conducted in the absence of any commercial or financial relationships that could be construed as a potential conflict of interest.

## Appendix

**Appendix A:** Questionnaire.

**Variables of the study**	**λ**	**CR**	**AVE**	**α**
**Entertainment** (Park et al., 2009) [1- strongly disagree to 5- strongly agree]				
E1: Sharing COVID-19 information through WhatsApp is entertaining for me.	0.76	0.82	0.61	0.82
E2: Sharing COVID-19 information through WhatsApp is fun for me.	0.84			
E3: Sharing COVID-19 information through WhatsApp is exciting for me.	0.72			
**Information seeking** (Park et al., 2009) [1- strongly disagree to 5- strongly agree]				
IS1: I share COVID-19 information through WhatsApp to get useful information through other’s feedback.	0.72	0.81	0.59	0.72
IS2: I share COVID-19 information through WhatsApp to get other’s opinion through their feedback.	0.82			
IS3: I share COVID-19 information through WhatsApp to learn more about pandemic.	0.76			
**Status seeking** (Park et al., 2009) [1- strongly disagree to 5- strongly agree]				
SS1: I share COVID-19 information through WhatsApp, because I want others to perceive me as sociable.	0.71	0.74	0.50	0.70
SS2: I share COVID-19 information through WhatsApp, because I want others to perceive me as knowledgeable.	0.65			
SS3: I share COVID-19 information through WhatsApp, because I want others to perceive me as valuable.	0.74			
**Socializing** (Park et al., 2009) [1- strongly disagree to 5- strongly agree]				
SO1: I share COVID-19 information through WhatsApp to share something with others.	0.73	0.77	0.53	0.76
SO2: I share COVID-19 information through WhatsApp to stay in touch with people I know.	0.77			
SO3: I share COVID-19 information through WhatsApp to feel like I belong to a community.	0.69			
**Habitual Diversion** (Malik et al., 2016) [1- strongly disagree to 5- strongly agree]				
HD1: I share information through WhatsApp as it is a part of my routine.	0.67	0.76	0.54	0.71
HD2: I share information through WhatsApp as it is one of my habits.	0.72			
HD3: I cannot stop myself sharing information through WhatsApp.	0.79			
**Attitude Toward Behavior** (Chen et al., 2018)				
ATB1: For me, sharing information about COVID-19 through WhatsApp is: (1-Bad to 5-Good)	0.69	0.80	0.57	0.79
ATB2: For me, sharing information about COVID-19 through WhatsApp is: (1-Foolish to 5-Wise)	0.80			
ATB3: For me, sharing information about COVID-19 through WhatsApp is: (1-Harmful to 5-Beneficial)	0.77			
**Perceived Behavioral Control** (Zhao et al., 2016) [1- strongly disagree to 5- strongly agree]				
PBC1: I think it’s easy for me to share COVID-19 information though WhatsApp.	0.75	0.83	0.62	0.83
PBC2: I am confident enough, if I want to share COVID-19 information though WhatsApp, I can.	0.80			
PBC3: I have time, resources and knowledge to share COVID-19 information though WhatsApp.	0.81			
**Subjective Norms** (Cheung and To, 2016) [1- strongly disagree to 5- strongly agree]				
SN1: My friends would think I should share information about COVID-19 through WhatsApp.	0.76	0.81	0.58	0.70
SN2: My family would think I should share information about COVID-19 through WhatsApp.	0.73			
SN3: My colleagues would think I should share information about COVID-19 through WhatsApp.	0.81			
**Behavioral Intention** (Zhao et al., 2016) [1- strongly disagree to 5- strongly agree]				
BI1: I will verify the authenticity of information about COVID-19 before sharing through WhatsApp.	0.80	0.83	0.63	0.83
BI2: I am willing to refute rumors about COVID-19 on WhatsApp.	0.83			
BI3: I will make efforts to refute rumors about COVID-19 on WhatsApp.	0.73			
**Actual Behavior** (Oh et al., 2013) [1- strongly disagree to 5- strongly agree]				
AB1: During pandemic (COVID-19), I transmitted information through authentic institutions.	0.78	0.87	0.68	0.86
AB2: During pandemic (COVID-19), I had only transmitted information with external source interlinkage.	0.84			
AB3: During pandemic (COVID-19), I had confirmed the authenticity of information before sharing through WhatsApp.	0.85			
**Norms of Reciprocity** (Pai and Tsai, 2016) [1- strongly disagree to 5- strongly agree]				
NOR1: I would feel an obligation to share COVID-19 information with others to help them be informed.	0.76	0.83	0.62	0.71
NOR2: When I receive COVID-19 information from others through WhatsApp, feel it right to share out to help others.	0.79			
NOR3: I would feel an obligation to spare time from my schedule to share COVID-19 information within WhatsApp community, if it needed that information.	0.81			
